# *HMA4* expression in tobacco reduces Cd accumulation due to the induction of the apoplastic barrier

**DOI:** 10.1093/jxb/ert471

**Published:** 2014-01-13

**Authors:** Oskar Siemianowski, Anna Barabasz, Maria Kendziorek, Anna Ruszczyńska, Ewa Bulska, Lorraine Elizabeth Williams, Danuta Maria Antosiewicz

**Affiliations:** ^1^University of Warsaw, Faculty of Biology, Institute of Experimental Plant Biology and Biotechnology, Miecznikowa str. 1, 02-096 Warszawa, Poland; ^2^University of Warsaw, Faculty of Chemistry, Pasteura str. 1, 02-093 Warszawa, Poland; ^3^University of Southampton, Centre for Biological Sciences, Building 85, Highfield, Southampton SO17 1BJ, UK

**Keywords:** AtHMA4, cadmium, microarray, P1BATPase, tobacco, transformation.

## Abstract

Ectopic expression in tobacco (*Nicotiana tabacum* v. Xanthi) of the export protein AtHMA4 (responsible in *Arabidopsis* for the control of Zn/Cd root to shoot translocation) resulted in decreased Cd uptake/accumulation in roots and shoots. This study contributes to understanding the mechanisms underlying this Cd-dependent phenotype to help predict the consequences of transgene expression for potential phytoremediation/biofortification-based strategies. Microarray analysis was performed to identify metal homeostasis genes that were differentially expressed in roots of Cd-exposed *AtHMA4*-expressing tobacco relative to the wild type. It was established that down-regulation of genes known to mediate Cd uptake was not responsible for reduced Cd uptake/accumulation in *AtHMA4* transformants. The transcript levels of *NtIRT1* and *NtZIP1* were higher in transgenic plants, indicating an induction of the Fe and Zn deficiency status due to *AtHMA4* expression. Interestingly, upon exposure to Cd, genes involved in cell wall lignification (*NtHCT*, *NtOMET*, and *NtPrx11a*) were up-regulated in transformants. Microscopic analysis of roots demonstrated that expression of *AtHMA4* caused an induction of cell wall lignification in the external cell layers that was accompanied by enhanced H_2_O_2_ accumulation. Further study showed that the concentration of other elements (B, Co, Cu, Ni, Mo, and Zn) was reduced in *AtHMA4* transformants in the presence of Cd. In conclusion, due to ectopic expression of 35S::*AtHMA4*, the physical apoplastic barrier within the external cell layer developed, which is likely to be responsible for the reduction of Cd uptake/accumulation.

## Introduction

Cadmium (Cd) is a heavy metal that is widespread in the environment, occurring both naturally and as a result of anthropogenic activities (Clemens *et al.*, 2013). It is highly toxic to living organisms, even at low concentrations. Although it is a non-essential element it is taken up by plants from the soil and enters the food chain, posing a threat to human health (Williams and Salt, 2009). To reduce Cd levels in contaminated soil, the process of phytoremediation, which is a plant-based technology, is under development. There are various types of phytoremediation, and phytoextraction has potential for pollutants such as Cd; this involves uptake and accumulation of the pollutant into the plant biomass from the environment. It is predicted that plant species useful for metal phytoremediation/phytoextraction would have the ability to take up the metal from the soil, and efficiently translocate and accumulate it in the shoot, which could then be easily harvested. Translocation of Cd (and other heavy metals) from the roots to the shoots is under tight control. In the majority of plant species (except metal hyperaccumulators) most of the metal is stored preferentially in the root (Palmgren *et al.*, 2008; Zhao and McGrath, 2009). To overcome this barrier and enhance Cd accumulation in the shoots, which is important for successful phytoremediation, one approach is to transform high biomass species with genes responsible for Cd root to shoot translocation.

Cd is taken up and transported across plant membranes mainly by transporters for metals that are essential to plants such as the micronutrients Zn, Fe, and Mn, and the macronutrient Ca (Mills *et al*., 2005, 2012; Williams and Salt, 2009; Menguer *et al.*, 2013). Determining the specificity of transporters is thus important in understanding the nutrition of plants and also their susceptibility to toxic elements. The translocation of the micronutrient Zn, and also non-essential Cd to the shoots of *A. thaliana* is under the control of the *HMA2* and *HMA4* genes, which belong to the P_1B_-type ATPase family (Mills *et al*., 2003, 2005, 2010, 2012; Hussain *et al.*, 2004; Williams and Mills, 2005; Wong and Cobbett., 2009). Based on studies performed in several laboratories, it was concluded that both proteins are responsible for Zn and Cd xylem loading. The tissue-specific expression of *HMA2* and *HMA4* genes was detected mainly in the vasculature (Hussain *et al.*, 2004; Verret *et al.*, 2004). They encode plasma membrane-localized proteins (Verret *et al.*, 2004; Hussain *et al.*, 2004; Courbot *et al.*, 2007), and expression of *HMA4* in yeast and *Escherichia coli* established that it mediates export of Zn and Cd out of the cell (Mills *et al.*, 2003, 2005, 2012). Evidence for the involvement of both HMA2 and HMA4 in the control of Zn and Cd translocation to the shoots of *Arabidopsis* plants came from mutant analysis. It was shown that Zn and Cd levels in the shoots were severely reduced in the double *hma2hma4* mutant, and to a lesser extent in the single *hma4* mutant (Hussain *et al.*, 2004; Verret *et al.*, 2004; Wong and Cobbett, 2009; Mills *et al.*, 2010). It was also suggested that HMA4 plays a role in xylem loading of Zn and Cd, and hence in the control of translocation to shoots in *Arabidopsis halleri* and *Thlaspi caerulescens* (Bernard *et al.*, 2004; Papoyan and Kochian, 2004; Hanikenne *et al.*, 2008).

Since *HMA4* plays a crucial role in the control of Zn and Cd translocation to shoots, it was used to transform tobacco, a plant species suitable for phytoremediation/phytoextraction due to its high biomass and low nutritional requirements. Two *HMA4* genes have been expressed in tobacco: *AtHMA4* from *A. thaliana* under the constitutive *Cauliflower mosiac virus* (CaMV) 35S promoter (Siemianowski *et al.*, 2011); and *AhHMA4* from *A. halleri* (Zn/Cd hyperaccumulator) under its native promoter (Barabasz *et al.*, 2010). In transgenic tobacco plants expressing either 35S::*AtHMA4* or *AhHMA4p::AhHMA4*, Zn translocation to the shoots was facilitated, although in a Zn supply-dependent manner. The results indicated an interplay between transgene activity and the different molecular background of the transformed plants at a range of Zn levels in the medium. However, Cd levels in the shoots, as well as in the roots were unexpectedly reduced in *HMA4* transformants. The results obtained suggested substantial modifications of the host plant transcriptome and metabolome due to the expression of *HMA4*, which contributed to the generation of the phenotype. Understanding the mechanisms underlying these modifications is important in planning future strategies for biotechnology purposes. Engineering the root/shoot metal distribution is crucial not only for phytoremediation but also for biofortification. It is commonly said that phytoremediation and biofortification are ‘two sides of the same coin’ (Guerinot and Salt, 2001). For phytoremediation/phytoextraction purposes, enhanced translocation of polluting heavy metals is desired, whereas for biofortification it is important to enhance translocation of beneficial micronutrients such as Zn and Fe, while limiting the accumulation of toxic metals such as Cd in the edible parts. Therefore, understanding how plants accumulate and store heavy metals will help in engineering their distribution in the appropriate plant parts depending on the ultimate objective (phytoextraction/biofortification).

This study aims to understand the processes that were modified in tobacco as a result of the expression of 35S:*AtHMA4*, which ultimately leads to decreased Cd uptake and translocation to shoots. Clarifying the underlying mechanisms contributes to developing strategies aimed at producing plant-derived food with an enhanced level of micronutrients and reduced content of non-essential toxic metals such as Cd. It is also relevant to strategies aimed at producing tobacco with low Cd-containing leaves, which is of great importance for tobacco producers and smokers. Here it is demonstrated that ectopic expression of *AtHMA4* in tobacco alters the physical apoplastic barrier within the root external cell layer, contributing to a reduction in Cd accumulation.

## Materials and methods

### Plant material and general growth conditions

Experiments were performed on wild-type tobacco (*Nicotiana tabacum* v. Xanthi) and two homozygous lines (nos 5 and 9) of transgenic tobacco expressing *AtHMA4* from *Arabidopsis thaliana* (Siemianowski *et al.*, 2011). Plants were cultivated in a growth chamber, at 23/16 °C day/night temperatures, 40–50% humidity, with a 16h photoperiod at a quantum flux density (PAR) of 250 μmol m^–2^ s^–1^ using fluorescent Flora tubes.

Seeds were surface sterilized in 8% sodium hypochloride and germinated on 1/4 Knop’s medium (Barabasz *et al.*, 2013) supplemented with 2% (w/v) sucrose and solidified with 1% agar on Petri dishes positioned vertically. Two-week-old seedlings were transferred to 2 litre pots containing 1/4 Knop’s liquid medium for 2 weeks. The nutrient solution was renewed every 4 d. Then plants of the same size were transferred to basic nutrient solution supplemented with 0.25 μM Cd (added as CdCl_2_) for 4 d. In parallel, control plants were cultivated on basic medium without Cd. At the end of the experiments, roots of 4.5-week-old plants were collected for the following purposes: (i) to examine transcriptional profiles by microarray analysis; (ii) to determine the expression levels of selected genes; (iii) to determine the lignin level and tissue localization; (iv) to assess the H_2_O_2_ level and tissue localization; and (v) to perform the ionomic profiles.

### RNA isolation

Total RNA was extracted with the use of an RNeasy Plant Mini Kit (Qiagen, Hilden, Germany) according to the manufacturer’s recommendations, followed by DNase I digestion (Invitrogen). All RNA samples were quantified at *A*_260_ using a Nanodrop spectrophotometer ND100 (Nanodrop, Wilimington, DE, USA). The quality of RNA used for microarray analysis was checked using an Agilent 2100 Bioanalyser (Agilent Technologies, Santa Clara, CA, USA), according to the manufacturer’s instructions.

### Microarray analysis

Microarray-based analysis was performed to compare root gene expression profiles in tobacco expressing 35S-*AtHMA4* (line no. 9) and wild-type plants, which were grown in the presence of 0.25 μM Cd. Three independent experiments were performed. At the end of each experiment (for details, see the section ‘Plant material and general growth conditions’), roots from six plants (excluding one-third of the distance from their base) were pooled, frozen in liquid nitrogen, and stored until RNA isolation. However, from each individual plant, only half of the root system was collected and pooled for the microarray experiment. The other half was collected separately, dried, and used for analysis of Cd concentration. In addition, shoots from these plants were also collected for determination of Cd concentration. Total RNA isolated from three batches of roots was used for three independent microarray analyses.

The Affymetrix ATCTOBa520488 containing 40 642 tobacco unigenes was used to compare the expression profiles of *AtHMA4*-expressing tobacco and the wild-type. RNA labelling and hybridization to the tobacco cDNA microarray Affymetrix ATCTOBa520488 (Edwards *et al.*, 2010) was conducted in the Nottingham Arabidopsis Stock Centre (NASC, http://arabidopsis.info/) (Craigon *et al.*, 2004). Synthesis of the complementary RNA [labelling of RNA samples was performed by use of the Affymetrix 3’ IVT-Express kit (Affymetrix UK Ltd., High Wycombe, UK)] followed standard Affymetrix protocols.

Data sets contained in the (.CEL) files were normalized according to the Robust Multi-array Average (RMA) approach using DChip software (Li and Wong, 2001). Further analysis was performed with the same software (Li and Wong, 2001; Li, 2008). Gene expression was considered as up- or down-regulated if the transcript level showed a minimum of a 1.3-fold change, with an e-value of 0.05 and 200 permutations. Annotations conducted based on Edwards *et al.* (2010) were supplemented with annotations for the unigenes taken from the BLASTX (NCBI) hit of *N. tabacum* (*e*-value <1×10^–1^) non-redundant proteins from GenBank. Functional categorization was performed using Gene Ontology (GO) analysis tools available at TAIR http://www.arabidopsis.org/portals/genAnnotation/functional_annotation/go.jsp] and AmiGO–GO Term Enrichment (http://amigo.geneontology.org/cgi-bin/amigo/term_enrichment). Due to a lack of such tools for tobacco, functional categorization was performed using the best hit of *A. thaliana* genes homologous to identified *N. tabacum* sequences that were differentially expressed in transgenic plants relative to the wild type.

Microarray data have been released onto the NASC Arrays database: http://affy.arabidopsis.info/narrays/experimentpage.pl?experimentid=699.

The accession number for the EMBL sequence database is GSE47037

### Real-time PCR analysis of gene expression

For confirmation of the microarray expression profiling data, quantitative PCR was carried out for a chosen subset of genes. In addition to genes identified by microarray analysis, expression levels of other selected metal homeostasis genes were estimated. Expression analysis was performed using total RNA isolated from roots of transgenics (line nos 5 and 9) and wild-type plants grown on 1/4 Knop’s medium with and without Cd.

The cDNA was synthesized in a 20 μl reaction volume containing 1 μg of the total RNA and oligo d(T)_18_ primers according to procedures described by Wojas *et al.* (2007). The cDNA was used as a template for real-time PCR using Platinum SYBR Green qPCR superMix-UDG (Invitrogen) according to the manufacturer’s recommendations. The MyiQ(™)2 cycler (Bio-Rad Laboratories Inc., Hercules, CA, USA) was used.

Primer pairs for each gene were designed using BLAST-PRIMER i PRIMER software (http://www.ncbi.nlm.nih.gov/tools/primer-blast/) based on the corresponding sequences available in the database (http://www.ncbi.nlm.nih.gov/). The primer sequences are given in Table 1.

**Table 1.** Sequences of primers used for expression analysis

Gene name (accession no.)	Primer sequences	Product size (nt)
*CAX3* (EB428626)	F: 5′-GCAATTCTTGCTCTGACGCA-3′	320
	R: 5′-AGGTACCCGAGGTAGGCAAT-3′	
*NtHCT* (DW003425)	F: 5′-TCTCATCCCAACAGCAGACA-3′	113
	R: 5′-CAAAAGGGCAACCAGTTCCA-3′	
*NtOMT I* (EB444811)	F: 5′-TGTTGGAGGTGCACTTGGTT-3′	114
	R: 5′-CCGGGATAAGCAGGAGCAAT-3′	
*NtVCaB42* (EB426333)	F: 5′-CATGGGAAAGAGATGCTCGC-3′	123
	R: 5′-AGGCTCTTCTTGCACCCAAA-3′	
*NtZIP1* (AB505626)	F: 5′-TGGTGGCTCAGTCTGGAGAT-3′	94
	R: 5′-CGAAGGAGCTCAGAACTGGAA-3′	
*NtIRT1* (AB263746)	F: 5′-ACTTACGAGGAGAACAGCCC-3′	73
	R: 5′-TCAGAAGGCCAGCAGATGAC-3′	
*Ntprx11a* (BAA82306)	F: 5′-AGGAGAGAAAAGGGCTGCAC-3′	103
	R: 5′-AGAAACAACACCAGGGCACA-3′	
*NtEF1a* (D63396)	F: 5′-GCTCCCACTTCAGGATGTGTA-3′	60
	R: 5′-ACACGACCAACAGGGACAGT-3′	
*HMA-A* (HF675181)	F: 5′-ACAAAGTGCTCGGACACCAA-3′	155
	R: 5′-CTTCTCGGTTGCAGAGTCCT-3′	
*HMA-B* (HF937054)	F: 5′-ACAAAGTGCTCGGACACCAA-3′	155
	R: 5′-CTTCTCGGTTGCAGAGTCTA-3′	

All gene expression analysis was performed with at least three independent biological replicates. For each sample, reactions were set up in triplicate and means were calculated. The tobacco *NtEF1a* (*elongation factor 1a*) gene was used as reference gene/internal control and was amplified in parallel with the target gene, allowing gene expression normalization and providing quantification. The quantification of the relative transcript levels was performed using the comparative Ct (threshold cycle) method. Validation experiments were performed to test the efficiency of the target amplification and the efficiency of the reference amplification.

### Microscopic analysis of lignification of roots

Lignification of the roots was analysed at the cross-section using two approaches: (i) following staining with the fluorescent dye safranin-O; and (ii) based on lignin autofluorescence. Analysis was performed using an NICON A1 multiphoton Ti:Sapphire confocal laser scanning microscope. Roots of wild-type and *AtHMA4*-transformed tobacco (line nos 5 and 9) were grown as described above and used to make hand sections as follows. Root pieces of 3cm long cut from the root tip were embedded in cooled 1.8% agar to solidify further in blocks. Handmade consecutive cross-sections were made at a distance of 2cm from the root tips (thickness approximately <0.5mm) and analysed for the presence of lignin according to standard procedures using safranin-O and the analysis of lignin autofluorescence (De Micco and Aronne, 2007; Bond *et al.*, 2008). Sections were stained in 0.5% safranin-O dissolved in 50% ethyl alcohol (v/v) for 2min; the dye was washed out with ethanol, then with water, and sections were examined by confocal microscopy. All experiments were repeated 3–4 times. For each experimental condition, at least three plants were analysed, from each plant at least 10 roots were taken, and from each root ~20 consecutive sections were made.

### H_2_O_2_ histochemical staining at the tissue level

Hydrogen peroxide accumulation was visualized with 3,3’-diaminobenzidine (DAB) according to Wojas *et al.* (2008). Roots of transgenic plants (line nos 5 and 9) and the wild type grown as described above were used for analysis. Root pieces of ~3cm long cut from the root tips were immersed in 5mM DAB solution (pH 3.8), vaccum infiltrated (at –0.04MPa) for 15min to increase solution penetration, and exposed to DAB for a further 8h in darkness. Afterwards roots were embedded in the agar blocks (as described above). Handmade cross-sections were analysed under a light microscope (Zeiss). Brown deposits indicate the localization of hydrogen peroxide and the intensity of staining indicates its amount. All experiments were repeated 3–4 times. For each experimental condition, at least three plants were analysed, from each plant at least 10 roots were taken, and from each root ~20 consecutive sections were made.

### Elemental profiles

Concentrations of Cd, Al, B, Co, Cu, Fe, Ni, Mo, Zn, and Ca were assessed in *AtHMA4*-transformed plants (line nos 5 and 9) and the wild type grown as described above. At the end of the Cd exposure, roots and shoots of control and Cd-treated plants were separated. Roots were washed as described in Wojas *et al.* (2009). Roots and shoots were dried in an oven at 55 °C until reaching constant weight. Dried samples were powdered and wet ashed as described by Zabłudowska *et al*. (2009). Briefly, acid digestion was performed in a mixture of 65% HNO_3_ and 30% H_2_O_2_ (9:1) for 15min in a closed system microwave mineralizer (Milestone Ethos 900, Milestone, Bergamo, Italy). Concentrations of elements were determined by inductively coupled plasma mass spectrometry (ICP-MS, Model Elan 9000; Perkin Elmer Sciex, Thornhill, Ontario, Canada). The analysis of the certified reference material SWR-2 Trace Elements (The National Institute of Standards and Technology, NIST, USA) yielded results fitting the range of certified recommended values.

Statistical significance was evaluated at the 0.05 probability level using the Student’s *t*-test. The data are expressed as means ±SD. Analysis was performed with Excel 2003 for Windows. Experiments were repeated three times.

## Results

### Microarray analysis determining the effects of AtHMA4 expression in tobacco

AtHMA4 is an export protein responsible for Zn and Cd root to shoot translocation in *Arabidopsis* (Mills *et al.*, 2003, 2005; Hussain *et al.*, 2004; Verret *et al.*, 2004, Wong and Cobbett, 2009). However, when expressed in tobacco it resulted in a reduction in Cd accumulation and it was suggested that this may result from a modification of endogenous gene expression (Siemianowski *et al.*, 2011). To explore this further, microarray analysis was conducted to screen for metal homeostasis genes that were differentially expressed in tobacco transformed with *AtHMA4* relative to the wild type. Following treatment, the root system of each plant was split so that half could be used for RNA extraction for microarray studies and half for Cd concentration determinations. The Cd content of shoot material was also determined. In agreement with the results presented by Siemianowski *et al.* (2011), Cd concentrations in the roots and the shoots were significantly lower in *AtHMA4* transformants compared with the wild type (Supplementary Fig. S1 available at *JXB* online).

**Fig. 1.**
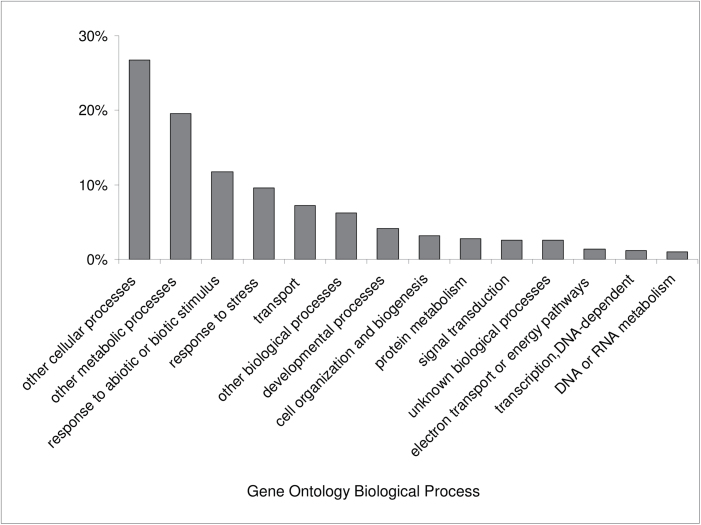
Gene Ontology distribution of the gene groups identified by the comparative microarray approach. Genes were identified by microarray analysis as differentially expressed in roots of 4.5-week-old *AtHMA4*-expressing tobacco (line 5) as compared with the wild type (WT) grown in the presence of 0.25 μM Cd for 4 d. Tobacco genes were classified by GO of biological processes using the *Arabidopsis* sequences from the TAIR database.

Based on the total number of genes analysed, *AtHMA4* transformants exposed to 0.25 μM Cd showed ~0.3% change in transcript abundance compared with the wild type. In the Cd-treated roots of *AtHMA4*-expressing tobacco, 123 genes were differentially expressed; 56 genes were up-regulated and 67 down-regulated (Supplementary Table S1 available at *JXB* online). Out of 123 differentially expressed genes, 18 showed a >2-fold difference, whereas for the remaining 105 genes the fold change was between 1.3 and 2. It was decided to include genes with these lower expression differences in the analysis to avoid overlooking important mechanisms underlying the generation of the Cd-dependent phenotype in *AtHMA4* transformants. The relative distribution (%) of differentially expressed genes classified by GO of biological processes (using the *Arabidopsis* sequences from the TAIR database) are shown in Fig. 1. A relatively large group of genes were attributed to categories that may be considered important in the regulation of a plant’s response to Ca such as ‘response to abiotic and biotic stimulus’, ‘response to stress’, and ‘transport’.

The aim of the microarray experiment was to reveal metal homeostasis genes differentially expressed in *AtHMA4* transformants, which might be responsible for the reduction of Cd uptake and translocation to shoots. A selection of these were identified for further analysis (Table 2). First, taking into account that Cd is translocated across biological membranes through pathways for essential minerals, primarily for Fe, Zn, and Ca, genes known from their involvement in metal transport were a primary focus. Only two metal transporters with significant differences in transcript levels in Cd-exposed *AtHMA4* transformants relative to the wild-type plants were identified. The first was *NtZIP1* encoding a putative tobacco Zn transporter. The second was a sequence homologous to *A. thaliana CAX3* (AT3G51860), a putative tobacco Ca transporter. Furthermore, the vacuole-associated annexin VCaB42 calcium-dependent membrane-binding protein homologous to *A. thaliana* ANNAT4 was also selected. It is thought that annexins form Ca^2+^-permeable channels (Gorecka *et al.*, 2007; Huh *et al.*, 2010) and thus could be involved in Cd transport as well. In addition, three up-regulated genes involved in cell wall lignification were selected (*NtHCT*, *NtOMET*, and *NtPrx11a*), as it is known that lignification restricts apoplastic Cd translocation and therefore might contribute to decreased metal accumulation (Lux *et al.*, 2011).

**Table 2.** List and description of genes up- and down-regulated (±1.3-fold) in roots of AtHMA4-expressing tobacco following a 4 d exposure to 0.25 μM Cd (analysed by microarray)

GenBank accession	*N. tabacum* best hit (blast x) accession no.	Sequence definition of *N. tabacum* best hit (NCBI)	Fold change	*P*-value	*A. thaliana* best hit (blastx) accession no.	Sequence definition of *A. thaliana* best hit (NCBI)	Max. identity to *A. thaliana* best hit (NCBI)
Metal homeostasis							
EB428626	Unknown	Unknown	2.11	0.0105	AT3G51860	CAX3 (cation exchanger 3); cation:cation antiporter	68%
BP528234	AB505626	Metal transporter NtZIP1	2.73	0.0177	AT3G12750	AtZIP1 (ZINC TRANSPORTER 1 PRECURSOR)	64%
Response to chemical stimulus							
EB426333	AAD24540	Vacuole-associated annexin VCaB42 calcium-dependent membrane-binding protein	–1.35	0,0228	AT2G38750	ANNAT4 (ANNEXIN ARABIDOPSIS 4); calcium ion binding/calcium- dependent phospholipid binding	49%
Cell wall/lignin biosynthesis							
DW003425	Q8GSM7	Hydroxycinnamoyl transferase, transferase family protein. Acyltransferase involved in the biosynthesis of lignin NtHCT	1.33	0.0248	AT5G48930	Hydroxycinnamoyl- CoA shikimate/quinate hydroxycinnamoyl transferase	36%
EB444811	CAA52461	Catechol *O*-methyltransferase, involved in lignin biosynthesis NtOMT I	1.55	0.0313	AT5G54160	AtOMET1, encodes a flavonol 3’-*O*-methyltransferase that is highly active towards quercetin and myricetin	52%
CQ809062	BAA82306	Peroxidase NtPrx11a (tpoxC1)	3.12	0.0008	AT5G05340	Peroxidase 52, cell wall peroxidase	66%

### Quantitative real-time PCR analysis

**Fig. 2.**
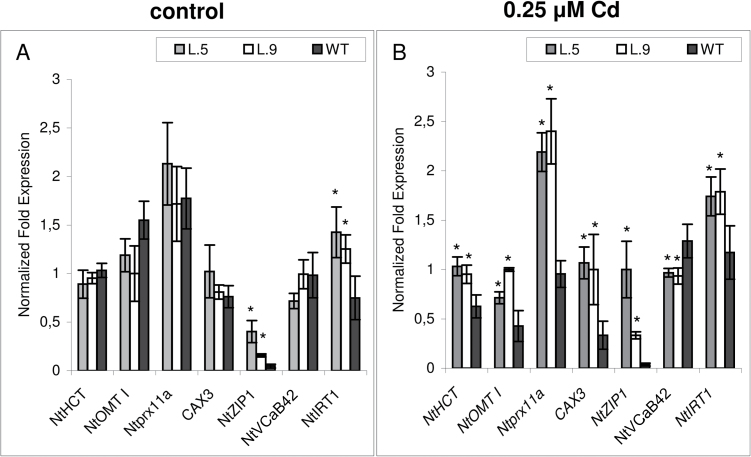
Confirmation of microarray results by quantitative real-time RT–PCR. The chosen genes identified by microarray analysis as differentially expressed in roots of 4.5-week-old *AtHMA4* expressing plants (lines 5 and 9) and in the wild type (WT) grown under control conditions (A) and in the presence of 0.25 μM Cd for 4 d (B). Moreover, the expression of *NtIRT1* was analysed. Normalized fold gene expression in plants grown under control conditions (A) and in plants grown in the presence of 0.25 μM Cd for 4 d (B). Values correspond to means ±SD (*n*=3); those significantly different from the WT (Student’s *t*-test) are indicated by an asterisk (*P* ≤ 0.05).

To validate the microarray results, differential expression of six selected genes listed in Table 2 was analysed by quantitative real-time reverse transcription–PCR (RT–PCR) (Fig. 2A, B). Analysis was performed for transgenic line no. 9 (used for microarray experiments) and also for line no. 5. Results shown in Fig. 2B confirm differences in the transcript abundance of all six genes identified by microarray experiments (*NtHCT*, *NtOMET*, *Ntprx11a*, *CAX3*, *NtZIP1*, and *NtVCaB42*). The expression of these six genes was also determined under control conditions without Cd (Fig. 2A) and, while no difference between transgenic and wild-type plants was detected for *NtHCT*, *NtOMET*, *NtPrx11a*, *CAX3*, and *NtVCaB42*, it was found that *NtZIP1* was up-regulated in *AtHMA4* transformants under control conditions. Thus, *AtHMA4* expression induced modifications of particular tobacco metal homeostasis genes not only upon Cd exposure but also in plants grown under control conditions.

It is known that Cd is transported through pathways specific for micronutrients, primarily for Zn, Fe, Mn, and also Ca, due to their low substrate specificity. Therefore it was thought that reduced Cd accumulation in transgenic plants could be due to down-regulation of genes potentially involved in Cd uptake; this was not the case and no such genes were detected based on microarray analysis. The only candidate gene identified that was possibly involved in the uptake of Cd is *NtZIP1*, but this was up-regulated (fold change 2.73) and not down-regulated. IRT1 is recognized as a major entry route for Cd to plant cells primarily under Fe-limiting conditions (Korshunova *et al.*, 1999). Since there were no probes present for *IRT1* in tobacco, the expression of *NtIRT1* was analysed by real-time RT–PCR (Fig. 2B). However, the expression of *NtIRT1* was higher in *AtHMA4* transformants than in the wild type in the presence of Cd; consequently, modified expression of this uptake gene does not appear to contribute to the reduction of Cd accumulation. In summary, these data demonstrate that it is rather unlikely that reduced Cd uptake in transgenic plants was due to the down-regulation of tobacco uptake genes.

**Fig. 3.**
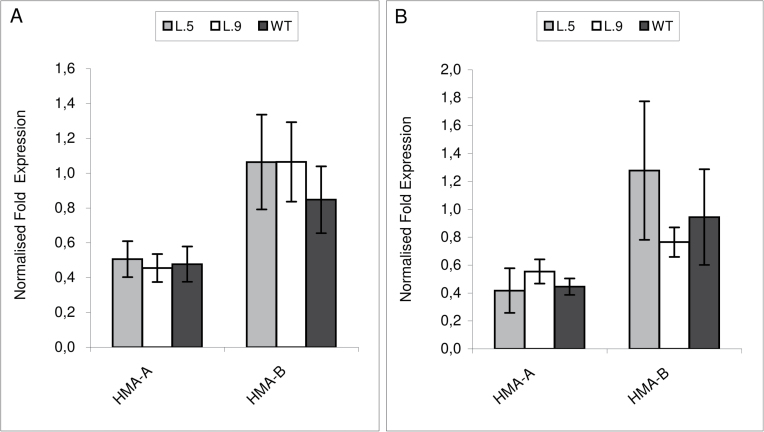
Expression analysis of tobacco *HMA4* by quantitative real-time RT–PCR. The level of expression of *NtHMA-A* and *NtHMA-B* was studied in roots of 4.5-week-old *AtHMA4*-expressing plants (lines 5 and 9) and in the wild type (WT) grown under control conditions (A), and in plants grown in the presence of 0.25 μM Cd for 4 d (B). Values correspond to means ±SD (*n*=3); those significantly different from the WT (Student’s *t*-test) are indicated by an asterisk (*P* ≤ 0.05).

Moreover, to examine if the expression of the endogenous tobacco *HMA4* was modified in the transgenic plants, the expression level of *NtHMA4* was compared in wild-type and transgenic tobacco grown under control conditions and upon exposure to 0.25 μM Cd. According to Dorlhac De Borne *et al.* (2012) there are two *HMA4* orthologues, designated as *HMA-A* and *HMA-B*. There were no probes for *HMA4* in the tobacco cDNA Affymetrix microarray; therefore, the expression was studied by real-time PCR only. As shown in Fig. 3, there was no difference in the expression level of both HMAs between the wild type and the tested transgenic lines. Thus, silencing of the endogenous *NtHMA4* as a potential cause of reduced Cd accumulation in the transgenic plants was excluded.

### Analysis of lignification and H_2_O_2_ accumulation at the tissue level

**Fig. 4.**
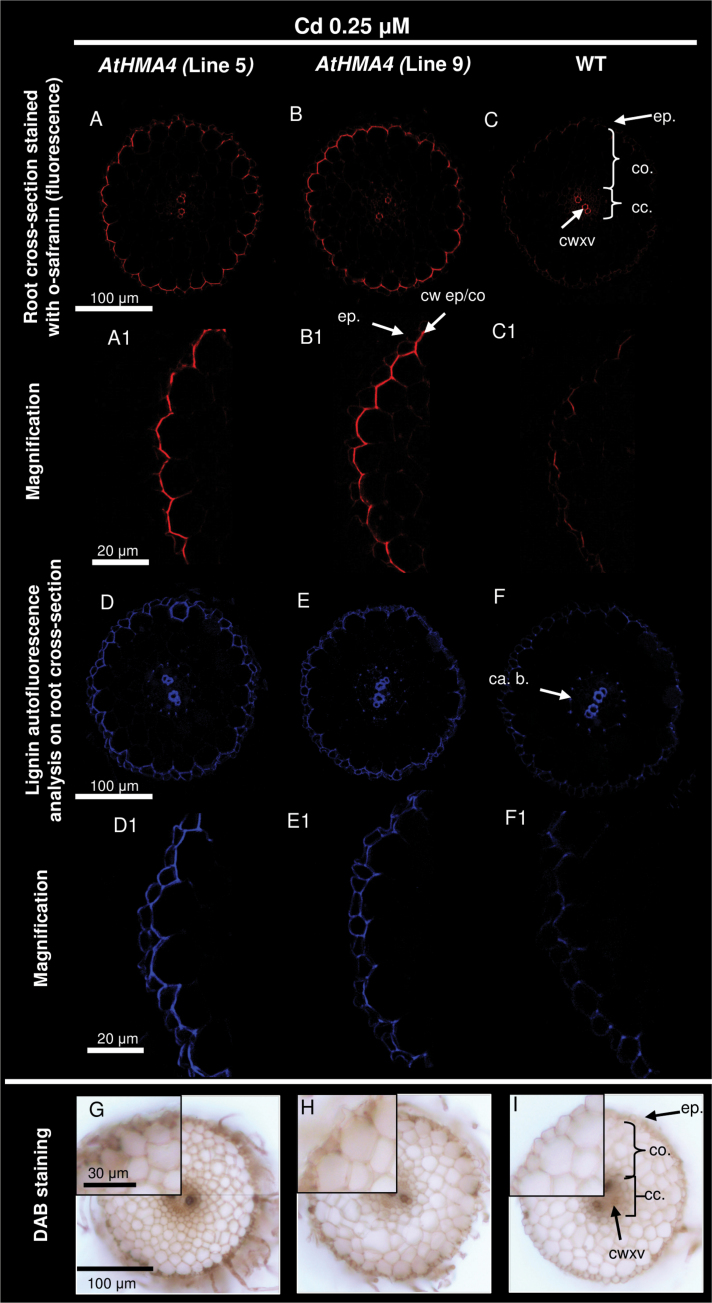
Lignification and H_2_O_2_ localization in roots of Cd-exposed plants. Analysis of lignification was performed under the confocal microscope with the use of safranin-O (A–C, A1–C1) and lignin autofluorescence (D–F, D1–F1). Cross-sections were made from roots of 4.5-week-old *AtHMA4*-expressing plants, lines 5 and 9 (A-B; A1-B1), and the wild type (WT) (C, C1) grown in the presence of 0.25 μM Cd for 4 d. ep., epidermis; co., cortex; cc., central cylinder; cwxv., cell wall of xylem vessel; cw ep/co, cell wall between epidermis and cortex; ca. b., Casparian band. H_2_O_2_ accumulation was assessed by the DAB reaction performed on cross-sections through roots of 4.5-week-old *AtHMA4*-expressing plants, lines 5 and 9 (G, H), and the WT (I) grown in the presence of 0.25 μM Cd for 4 d. H_2_O_2_ accumulation is indicated by dark-brown deposits.

**Fig. 5.**
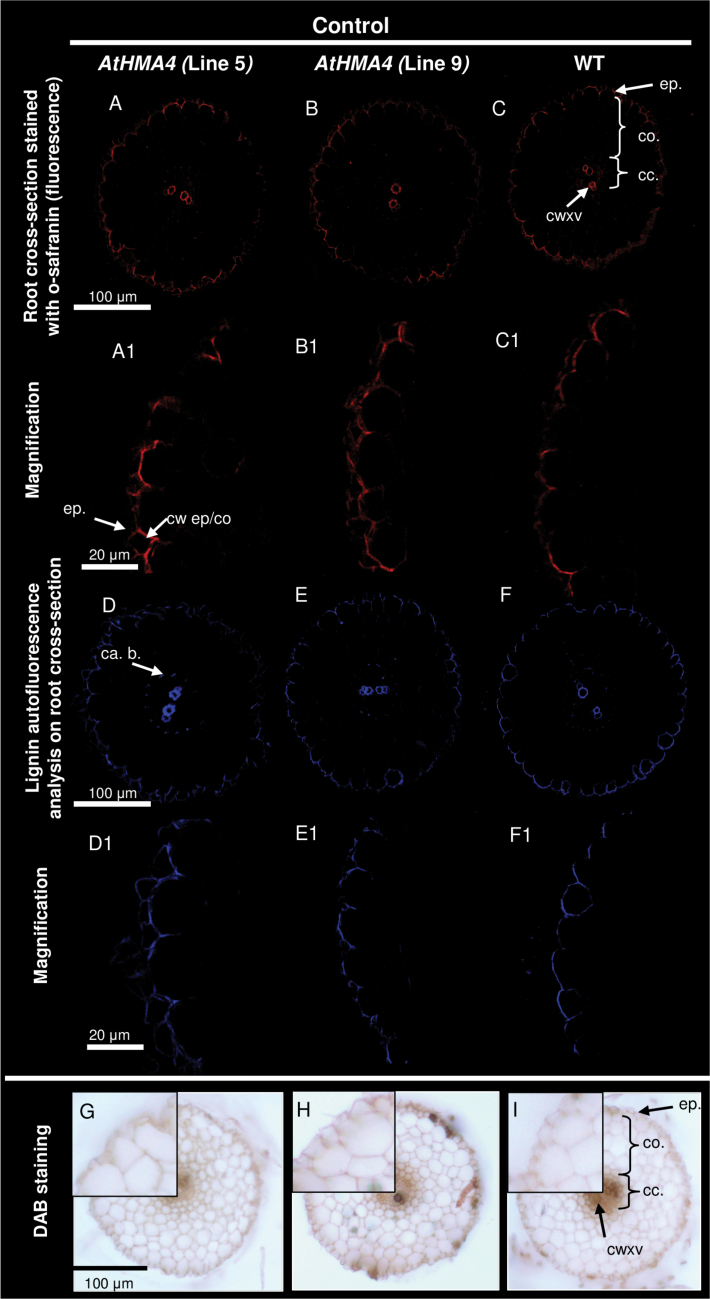
Lignification and H_2_O_2_ localization in roots of plants grown under control conditions. Analysis of lignification was performed under the confocal microscope with the use of safranin-O (A–C, A1–C1) and lignin autofluorescence (D–F, D1–F1). Cross-sections were made from roots of 4.5-week-old *AtHMA4*-expressing plants, lines 5 and 9 (A-B; A1-B1), and the wild type (WT) (C, C1) grown under control conditions. ep., epidermis; co., cortex; cc., central cylinder; cwxv., cell wall of xylem vessel; cw ep/co, cell wall between epidermis and cortex; ca. b., Casparian band. H_2_O_2_ accumulation was assessed by the DAB reaction performed on cross-sections through roots of 4.5-week-old *AtHMA4*-expressing plants, lines 5 and 9 (G, H), and the WT (I) grown under control conditions. H_2_O_2_ accumulation is indicated by dark-brown deposits.

Microarray experiments and real-time PCR expression analysis showed that in Cd-exposed *AtHMA4* transformants, *NtHCT* and *NtOMET*, known to be involved in the lignification of the cell wall (Whetten and Sederoff, 1995; Ibrahim *et al.*, 1998), were up-regulated (Fig. 2). It is known that Cd is translocated radially across the roots towards the xylem vessels preferentially through the apoplastic pathway; therefore, it is possible that lignification of the root tissues of transgenic plants could contribute to restricted translocation of this heavy metal and consequently to the reduced uptake and accumulation observed in both roots and shoots. To test this, the level of lignification of the cell walls was assessed by two methods: by lignin staining with safranin-O and by lignin autofluorescence analysis (Figs 4, 5). Higher fluorescence following safranin-O staining as well as lignin autofluorescence was detected in the cell walls of Cd-exposed *AtHMA4* transformants, between the epidermis and the first cortical layer (Fig. 4A-B, A1-B1, D-E, D1-E1). The difference between transgenic and wild-type plants grown without Cd was undetectable (Fig. 5A–F, A1–F1).

H_2_O_2_ is known to participate in lignin polymerization (Whetten and Sederoff, 1995), and elevated expression of *NtPrx11a*, which encodes a peroxidase, was detected in *AtHMA4* transformants (Fig. 2). Therefore, as a next step, quantification of tissue-specific accumulation of H_2_O_2_ using the DAB reaction was performed (Fig. 4G, H). Accumulation of H_2_O_2_ (visualized by brown deposits) in the roots of *AtHMA4* transformants was high in the apoplast between the epidermis and the first cortical layer, but this was not seen in wild-type roots (Fig. 4I). This is the same tissue localization where enhanced lignification occurred in transgenic plants (Fig. 5A-B, A1-B1, D-E, D1-E1). The results indicate that expression of *AtHMA4* leads to lignification of the cell walls in external cell layers of the roots, and this may restrict Cd uptake, translocation, and accumulation.

### Ionomic profile

**Fig. 6.**
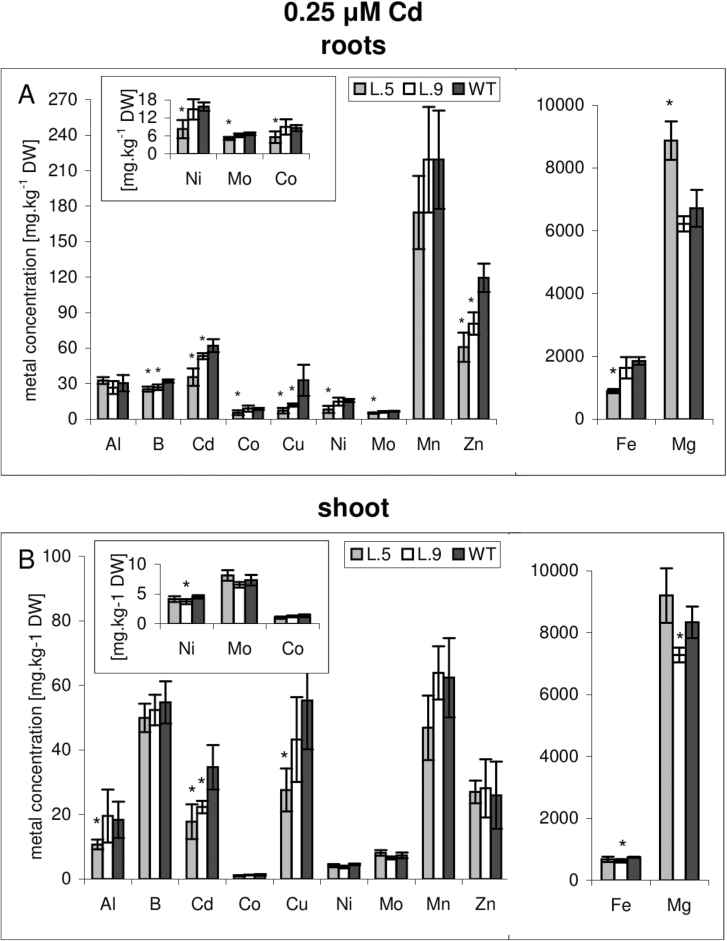
Ionomic profile of *AtHMA4*-expressing tobacco and the wild type (WT). Concentrations of Al, B, Cd, Co, Cu, Ni, Mo, Mn, Zn, Fe, and Mg in the roots (A) and shoots (B) of 4.5-week-old *AtHMA4*-expressing plants (lines 5, 9) and the WT exposed to 0.25 μM Cd for 4 d. Values correspond to arithmetic means ±SD (*n*=5). Values significantly different from the WT are highlighted by an asterisk (*P* ≤ 0.05) (evaluated by Student’s *t*-test)

**Fig. 7.**
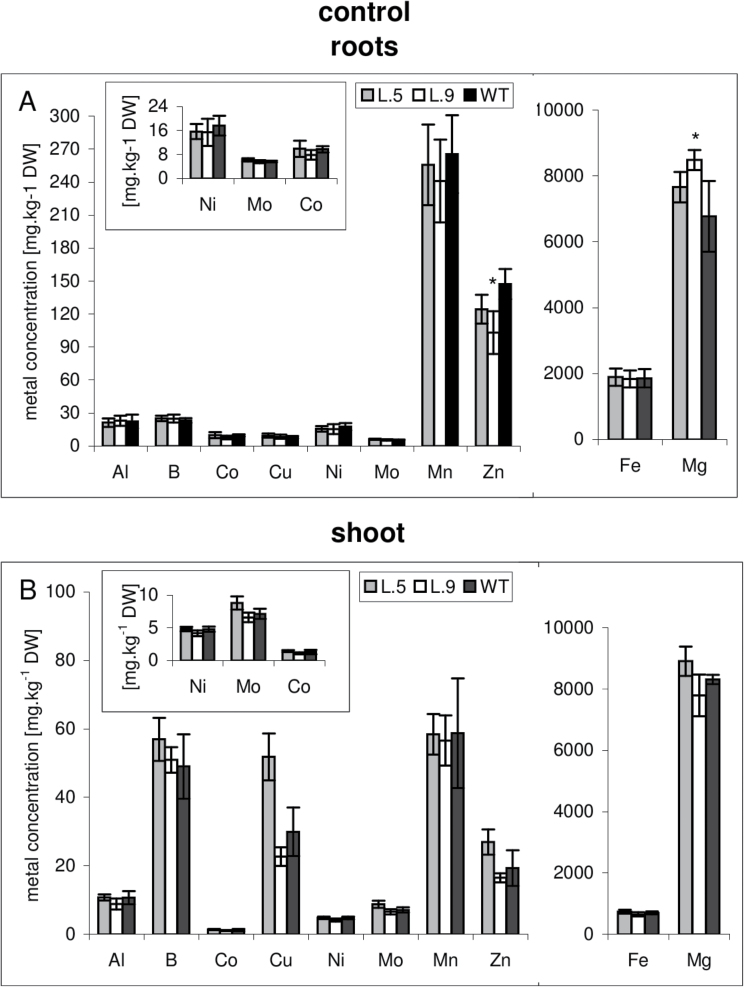
Ionomic profile of *AtHMA4*-expressing tobacco and the wild type (WT). Concentrations of Al, B, Cd, Co, Cu, Ni, Mo, Mn, Zn, Fe, and Mg in the roots (A) and shoots (B) of 4.5-week-old *AtHMA4*-expressing plants (lines 5, 9) and the WT grown under control conditions. Values correspond to arithmetic means ±SD (*n*=5). Values significantly different from the WT are highlighted by an asterisk (*P* ≤ 0.05) (evaluated by Student’s *t*-test).

The results obtained in the course of this study indicate that the apoplastic pathway across the root is blocked in *AtHMA4* transgenic plants by a lignification process, which could account for the reduced Cd accumulation observed in these plants. If that is the case, it was expected that the level of other elements in roots and shoots might be lower as well under these conditions. Therefore, the ionomic profiles of transgenic and wild-type plants grown in the presence of Cd and under control conditions were determined (Figs 6, 7). Upon Cd treatment, lower concentrations of Cd, B, Co, Cu, Ni, Mo, and Zn were detected in roots and to a lesser extent in shoots of transgenic plants (Fig. 6); under control conditions, no difference in the concentration of these elements was observed (Fig. 7). These results support the hypothesis that enhanced lignification of the cell walls within the external cell layer of the roots occurs in Cd-exposed plants heterologously expressing *35S:AtHMA4* and this contributes to the generation of a Cd-related phenotype of transformants (characterized by reduced Cd uptake/accumulation).

## Discussion

This study was aimed at understanding the mechanism of reduced Cd uptake and translocation to shoots observed in transgenic tobacco expressing *AtHMA4* (Siemianowski *et al.*, 2011, 2013). *AtHMA4* encodes the export protein responsible for loading of Zn and Cd into the xylem vessels, thus controlling root to shoot translocation in *Arabidopsis* (Hussain *et al.*, 2004; Verret *et al.*, 2004; Wong and Cobbett, 2009; Mills *et al.*, 2010). A genome-wide survey of genes differentially regulated in response to Cd in *AtHMA4*-transformed tobacco compared with the wild type was performed to identify modifications in major metal homeostatic pathways that could underlie the Cd-related phenotype of transformants.

It is known that in roots Cd is taken up primarily by the Fe, Zn, Mn, and Ca pathway (Clemens *et al.*, 2013). Therefore, it was expected that in transgenic plants the down-regulation of genes involved in uptake of micronutrients may take place. However, two potential metal uptake genes, *NtZIP1* and *NtIRT1*, were expressed at higher levels in Cd-exposed *AtHMA4* transformants (Table 2, Fig. 2; Supplementary Table S1 available at *JXB* online). Very little is known about the substrate specificity and physiological role of *NtZIP1*. The study by Sano *et al.* (2012) suggests that it is involved in Fe uptake since the *NtZIP1* transcript level was enhanced by high Fe (1mM) in BY-2 tobacco cells, and its expression in yeast cells enhanced iron uptake activity. The amino acid sequence of NtZIP1 is 61% identical to that of MtZIP3 of *Medicago truncatula*, which is able to restore growth of the *fet3fet4* yeast mutant on Fe-limited media and is down-regulated in leaves by Fe and Mn deficiency (López-Milán *et al*., 2004). On the other hand, its homologue *AtZIP1* from *A. thaliana* was shown to be localized at the tonoplast (Milner *et al.*, 2013), and to complement *zrt1zrt2* and *smf1* yeast mutants, indicating a capability for remobilizing both Zn and Mn from the vacuole. In *Arabidopsis*, *AtZIP1* is expressed in the central cylinder of the roots, and the transcript level abundance increased in response to Zn and Fe deficiency but not Mn and Cu deficiency (Grotz *et al.*, 1998; Milner *et al.*, 2013). Therefore, NtZIP1 may transport both Fe and Zn, and its up-regulation detected here (Fig. 2) may indicate an Fe and Zn deficiency status in the transgenic tobacco; this is supported by the lower Zn and Fe concentration in the roots of transformants exposed to Cd (Fig. 5A). This would be in agreement with the reported Fe and Zn deficiency status in *AhHMA4*-expressing tobacco and tomato (Barabasz *et al.*, 2011; Siemianowski *et al.*, 2013).

IRT1 (Iron-Regulated Transporter 1) is the main Fe^2+^ uptake gene operating primarily in the root epidermal cells (Connolly *et al.*, 2002). In *Arabidopsis*, in addition to Fe^2+^, it constitutes a major pathway for the influx of Zn^2+^ and Cd^2+^ (Vert *et al.*, 2002), and it is up-regulated not only in response to Fe deficiency but also in the presence of excess Zn (Becher *et al.*, 2004). In tobacco, *NtIRT1* expression is enhanced not only upon Fe deficiency conditions but also in the presence of Cd (Bovet *et al.*, 2006; Yoshihara *et al.*, 2006; Hodoshima *et al.*, 2007).

In this study, restriction of Cd accumulation in transgenic tobacco was accompanied by up-regulation of *IRT1* (Fig. 2). Since it is known that up-regulation of *IRT1* is an indicator of Fe deficiency status (Palmer and Guerinot, 2009), the results of this study indicate that the expression of *AtHMA4* in tobacco enhanced Cd-induced Fe deficiency. In summary, the up-regulation of *NtZIP1* and *NtIRT1* does not explain the reduction of Cd uptake/accumulation in roots and shoots of transgenic plants. It indicates, however, substantial disturbances not only in Zn status, but also in the Fe status, even though Fe is not a substrate for HMA4.

In *AtHMA4*-transformed tobacco, a sequence homologous to *CAX3* from *A. thaliana* was identified as highly up-regulated (Table 2, Fig. 2; Supplementary Table S1 available at *JXB* online). It is known that CAX transporters may also mediate Cd translocation across biological membranes (McAinsh and Pittman, 2009). They belong to the cation/H^+^ exchangers (CAXs) and coordinate the redistribution primarily of Ca, and also Cd, Mn and Zn (Hirschi *et al.*, 2000; Kamiya and Maeshima, 2004). In *Arabidopsis*, tonoplast-localized *CAX3* is known to be up-regulated by exogenous Ca (Hirschi, 1999; Shigaki and Hirschi, 2006), and is highly expressed in roots (Cheng *et al.*, 2005). It was suggested that AtCAX3 functions as a Ca transporter, although with weak activity (Cheng *et al.*, 2005). Interestingly, it has also been ascribed to mediating Ca^2+^/H^+^ transport during Na stress (Zhao *et al.*, 2008). The up-regulation observed here in transgenic tobacco of a *CAX3* homologue suggests that this sequence may play a specific role in tobacco in the regulation of ion homeostasis upon exposure to Cd. However, its role in generating *AtHMA4*-induced reduction of Cd accumulation does not seem to be of primary importance.

Differential regulation (down-regulation) in *AtHMA4* transformants was also identified for tobacco VCaB42 annexin (Table 2, Fig. 2; Supplementary Table S1 available at *JXB* online). Annexins are multifunctional lipid-binding proteins generally involved in linking Ca^2+^, redox, and lipid signalling to coordinate development with responses to biotic and abiotic stresses (Laohavisit and Davies, 2011). Their function in plants is poorly understood, and not much is known about the tobacco VCaB42 protein. It is reported as a tonoplast-associated protein that may play a role in vacuolar biogenesis for cell expansion (Seals and Randall, 1997). Recent studies indicate that annexins may cluster together at membranes to form a transport pathway (Laohavisit and Davies, 2011). Tobacco *VCaB42* shows high homology to *AnnAt4* from *A. thaliana*. *Arabidopsis AnnAt1* and *AnnAt4* interact with each other and regulate drought and salt stress responses (Huh *et al.*, 2010), and AnnAt1 was shown to form pH-sensitive ion channels in artificial lipid bilayers, again functioning in stress responses (Gorecka *et al.*, 2007). Moreover, it is known that expression of annexins is regulated by metals. For example, Zn affects *Thlaspi caerulescens* homologues of *AnnAt1/AtANN1* and *AnnAt2/AtANN2* (Tuomainen *et al.*, 2010), Cu affects *AnnAt3*/*AtANN3* and *AnnAt4*/*AtANN4* (Weber *et al.*, 2006), and Cd affects *AnnAt1*/*AtANN1* and pea root annexin abundance (Repetto *et al.*, 2003; Konopka-Postupolska *et al.*, 2009). However, the down-regulation of the tobacco *VCaB42* annexin homologue suggests that it is unlikely to be directly responsible for reduction of Cd uptake/accumulation in *AtHMA4*-expressing tobacco, but it is indicative of an important role for this protein in Cd responses, worthy of further study.

An important part of the study was to determine whether the expression of 35S::*AtHMA4* in tobacco resulted in modification of the expression level of endogenous *HMA4*, including its silencing. The involvement of HMA4 in the translocation of Cd to tobacco shoots was demonstrated by Dorlhac De Borne *et al.* (2012). These authors engineered tobacco with a reduced Cd concentration in shoots by silencing both *NtHMA-A* and *NtHMA-B* genes (Dorlhac De Borne *et al.*, 2012). In the present study, this possibility was excluded, as a comparative real-time analysis of the expression of both *HMA4* tobacco genes showed no difference between the wild type and transgenics cultivated under control conditions and in the presence of Cd (Fig. 3). In *Arabidopsis*, it has been shown that two P_1B_-ATPases, HMA2 and HMA4, contribute to the control of Zn and Cd root to shoot translocation (Wong and Cobbett, 2009). However, *HMA2* in tobacco has not been cloned yet, thus neither its sequence nor its function is known. In the future, it would be interesting to determine whether the function of HMA2/HMA4 is the same in tobacco as in *Arabidopsis*.

### *AtHMA4* expression induces development of an apoplasmic barrier for radial transport of water and elements in the external root layer

Several studies have demonstrated that exposure to Cd induces expression of genes involved in lignification in a number of plant species (Herbette *et al.*, 2006; Weber *et al.*, 2006; van de Mortel *et al*., 2008). Lignification of specific cell layers was proposed to contribute to restricting plant growth, but also to enhancing tolerance to Cd by reducing Cd uptake and translocation to shoots (Lux *et al.*, 2011). Using microarray-based analysis, it has been shown here that in *AtHMA4*-expressing tobacco with reduced Cd accumulation in roots and shoots, genes involved in lignification were up-regulated. Higher expression of *NtHCT* (hydroxycinnamoyl transferase), *NtOMT* I (*O*-methyltransferase), and *NtPrx11a* (peroxidase participating in lignin biosynthesis) (Whetten and Sederoff, 1995; Ibrahim *et al.*, 1998) indicated that upon Cd exposure the process of lignification was enhanced in *AtHMA4* transgenic plants. Further studies, performed using fluorescence microscopy, showed that in transgenic plants lignification did not occur across all root tissues, but specifically within cell walls between the epidermis and the first cortical layer (Fig. 4A-B, D-E). Furthermore, H_2_O_2_, which is known to be involved in lignin polymerization (Whetten and Sederoff, 1995), was enhanced in a similar location (Fig. 4G, H). These modifications were not detected in wild-type tobacco exposed to this concentration of Cd (Fig. 4C, F) nor in all the tested lines grown in control medium (Fig. 5).

It is known that Cd is translocated radially across root tissues primarily through the apoplastic pathway, and the exodermis and endodermis are considered as barriers to the apoplastic movement of numerous toxic heavy metals (Lux *et al.*, 2011). Therefore, it was hypothesized that lignification of an external layer in roots could contribute to the restriction of Cd accumulation in roots and consequently shoots of *AtHMA4*-expressing tobacco. However, if that was the case, a restriction of other elements may be expected. Indeed, the ionomic profile of transgenic plants (relative to the wild type) exposed to Cd for 4 d showed a reduction in nutrient elements, the most significant in roots, of Cu, Zn, and Cd, and to a lesser extent B, Co, Ni, Mo, and Fe. The reduction was weaker in shoots, which is likely to result from the compensative mechanisms regulating elemental long-distance translocation. In plants grown in control medium, there were no significant differences in the concentrations of the studied elements between control and *AtHMA4*-transformed lines (Fig. 7).

In summary, the microarray approach and biochemical and microscopic analysis led to the conclusion that expression of *AtHMA4* in tobacco induced the development of a physical barrier in the transport of elements towards the central cylinder by lignification of the cell walls in the outer layer of the roots. Its formation is likely to be the primary mechanism underlying the detected reduction of Cd accumulation by *AtHMA4*-transformed plants. However, it remains an open question as to why only one cell layer was lignified, and the nature of the underlying signal leading to this lignification. Lignification was induced in *AtHMA4* transgenic tobacco exposed to Cd within a cell layer that usually develops into the exodermis (Shufflebottom *et al.*, 1993; Enstone *et al.*, 2003). The exodermis constitutes an apoplasmic barrier to the uptake of water and ions, and it was shown that Cd accelerates its development. This was interpreted as an acclimatory response that restricts the apoplastic movement of Cd to the xylem and root to shoot translocation (Ma and Peterson, 2003; Seregin *et al.*, 2004; Seregin and Kozhevnikova, 2008; Lux *et al.*, 2011).

It was reported that the development of the exodermis is also regulated by other environmental stresses, including nutrient stresses, salinity, drought, and flooding/anoxia (Reinhardt and Rost, 1995; Enstone *et al.*, 2003; Karahara *et al.*, 2004; Meyer *et al.*, 2009). Its development contributes to restriction of apoplastic radial transport of both water and minerals at the root surface, influencing their loading to the xylem and aerial plant parts (Hose *et al.*, 2001; Enstone *et al.*, 2003; Baxter *et al.*, 2009). At the same time, it is involved in the movement of numerous substances from the root to the shoot, including abscisic acid, known as a root to shoot stress signal (Davis and Zhang, 1991). Thus, modulation of the permeability of the apoplast to water and solutes has far-reaching consequences for a plant’s response to environmental stresses.

In this study, lignification of the cell walls in the outermost cortical layer adjacent to the epidermis that was induced in transgenic plants might result from the combination of factors. One of these factors is overloading of the apoplast with Cd and Zn, which are substrates for AtHMA4 (Mills *et al.*, 2003, 2005; Hussain *et al.*, 2004; Siemianowski *et al.*, 2013). A higher concentration of metals in the apoplast across all tissues might contribute to the induction of the cell wall alterations; however, lignification was induced only within a specific cell layer. This unique response might be related to the difference in the ability of specific cell layers to undergo developmental modifications. It is probable that the lignification pathway is accessible for induction only in the cell layer that develops into the exodermis under development.

In conclusion, the ectopic expression of *AtHMA4* in tobacco does not mimic the physiological role it plays in *A. thaliana*. Instead, due to its export activity in all cells across the plant body, it disturbs the homeostasis of Zn, Fe, and also other metals. Consequently, the phenotype is generated as a response to these alterations. Interestingly, upon exposure to Cd, induction of a physical barrier in the apoplast in *AtHMA4* transgenic plants is likely to be responsible for the reduced accumulation of this metal.

## Supplementary data

Supplementary data are available at JXB online.

Figure S1. Cadmium concentration in roots and shoots of *AtHMA4*-expressing and wild-type plants.

Table S1. List of differently expressed genes between *AtHMA4*-expressing and wild-type tobacco exposed to 0.25 μM Cd.

Supplementary Data
