# Supplementary material for: HMA4 expression in tobacco reduces Cd accumulation due to the induction of the apoplastic barrier
Source: J Exp Bot. 2014 Jan 13;65(4):1125–39. doi: 10.1093/jxb/ert471 (PMC3935570; doi:10.1093/jxb/ert471)
Supplement: Supplementary Data [file supp_65_4_1125__index.html]

 AtHMA4 expression in tobacco reduces Cd accumulation due to the induction of the apoplastic barrier — HMA4 expression in tobacco reduces Cd accumulation due to the induction of the apoplastic barrier — Supplementary Data 

# *HMA4* expression in tobacco reduces Cd accumulation due to the induction of the apoplastic barrier

## Supplementary Data

Data files

**Files in this Data Supplement:**

- Supplementary Data - Supplementary Data
- Supplementary Data - Supplementary Data
