# Supplementary material for: HMA4 expression in tobacco reduces Cd accumulation due to the induction of the apoplastic barrier
Source: J Exp Bot. 2014 Jan 13;65(4):1125–39. doi: 10.1093/jxb/ert471 (PMC3935570; doi:10.1093/jxb/ert471)
Supplement: Supplementary Data [file supp_ert471_jexbot114355_file002.pdf]

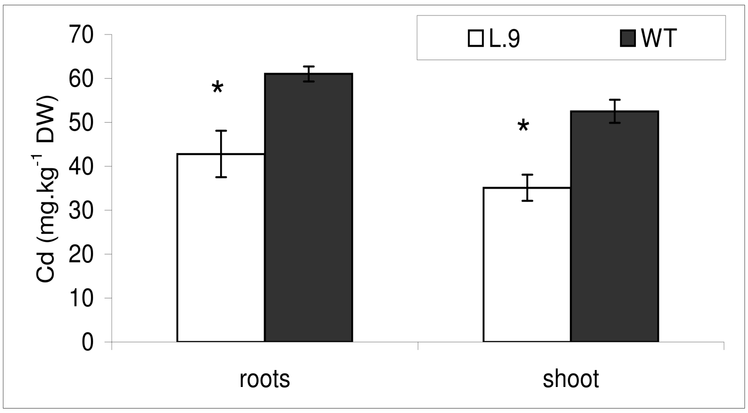

Figure 1S

Cadmium concentration in roots and shoots of *AtHMA4*-expressing and wild-type plants. 4.5-week old transgenic (lines 9) and wild type (WT) plants grown in hydroponics under control conditions were exposed for 4 days to 0.25 µM Cd. Values correspond to arithmetic means ± standard deviation (SD) (**n**=5). Values significantly different from WT are highlighted by an asterisk (**P**≤ 0.05) (evaluated by Student's **t**-test).
